# Epidemiological profile of anogenital lesions in 4,029 consultations at a Sexually Transmitted Infections Clinic in São Paulo, Brazil^؆^

**DOI:** 10.1016/j.abd.2025.501145

**Published:** 2025-07-10

**Authors:** Julia Aires Thomaz Maya, Kaique Arriel, Laura Stamato de Figueiredo, John Verrinder Veasey

**Affiliations:** a Dermatology Clinic, Hospital da Santa Casa de São Paulo, São Paulo, SP, Brazil; b Department of Dermatology, Universidade Santo Amaro, São Paulo, SP, Brazil; c Medical Degree, Centro Universitário Faculdade de Medicina do ABC, Santo André, SP, Brazil; d Discipline of Dermatology, Faculty of Medical Sciences, Santa Casa de São Paulo, São Paulo, SP, Brazil

*Dear Editor,*

Sexually transmitted infections (STI) are a public health problem due to their high prevalence in developing countries, such as Brazil, and their potential to cause morbidity.^1^, ^2^

A wide range of diseases of inflammatory and neoplastic etiology can affect the skin of the anogenital region, representing important differential diagnoses of STI.^3^, ^4^

Knowledge of diseases that affect the anogenital region is of utmost importance, since a delayed diagnosis exposes the patient to inadequate treatments, worsening their clinical condition and perpetuating the transmission of STI.^3^, ^4^ Data from the scientific literature on the epidemiology of these diagnoses are scarce, demonstrating the need for new population surveys.^5^

Therefore, the present study was conducted to identify the most frequent anogenital diagnoses in patients treated at the STI outpatient clinic of a tertiary hospital in the city of São Paulo, Brazil. This is a retrospective study analyzing the care provided between October 2012 and August 2024. Data on gender, age, and primary diagnosis were obtained from the institution's electronic medical record system.

During the evaluated period, 4,029 medical consultations were carried out due to anogenital complaints, 1,182 of which were female and 2,847 were male patients. The average number of consultations per year was 309.84 ± 192.08. Of the total number of patients, 2,992 were aged between 20 and 55 years. The average age of the patients was 42.77 ± 15.86 years, with a minimum age of 10 months (diagnosis of condyloma acuminatum) and a maximum of 92 years (condyloma acuminatum).

The main clinical diagnoses related to the consultations (Table 1) were condyloma acuminatum (CA) in 2847 consultations (70.8%) – shown in Fig. 1, followed by syphilis in 303 (5.7%), herpes simplex in 193 (4.4%), squamous cell carcinoma in 172 (4.3%) – shown in Fig. 2, lichen sclerosus et atrophicus in 98 (2.4%) – shown in Fig. 3, molluscum contagiosum in 80 (2%), eczema in 50 (1.2%) and lichen planus in 46 (1%). All cases of syphilis were primary syphilis (hard chancre, Follman's syphilitic balanitis, and “cord-like” lesion). Other diagnoses with less than 1% were grouped together, totaling 8.2% of the consultations.

**Table 1:** Distribution of the 4029 consultations by diagnosis and year.

Diagnosis	Number of consultations	%
Condyloma acuminatum	2841	70,5%
Syphilis	303	7,5%
Herpes simplex	177	4,4%
Squamous cell carcinoma	171	4,2%
Lichen sclerosus et atrophicus	89	2,2%
Molluscum contagiosum	79	2,0%
Eczema	50	1,2%
Lichen planus	40	1,0%
Others^a^	279	6,9%
Total	4029	100,0%

Year	Number of consultations	%
2012	18	0.4%
2013	266	6.6%
2014	702	17.4%
2015	524	13.0%
2016	451	11.2%
2017	498	12.4%
2018	365	9.1%
2019	430	10.7%
2020	192	4.8%
2021	219	5.4%
2022	197	4.9%
2023	149	3.7%
2024	104	2.6%
Total	4029	100.0%

a Candidiasis, Buschke-Lowenstein tumor, lymphogranuloma venereum, chancroid, lupia, vitiligo, pilonidal cyst, scar lesion, comedones, neuroma, plicoma, seborrheic keratosis, foreign body, varicocele, lymphangioma, Kaposi's sarcoma, hand-foot-and-mouth syndrome, melanocytic nevus, psoriasis, hidradenitis suppurativa, scabies, folliculitis, pharmacodermia, epidermodysplasia verruciformis, hemorrhoids, Behçet's disease, Paget's disease and Crohn's disease.

**Fig. 1:**
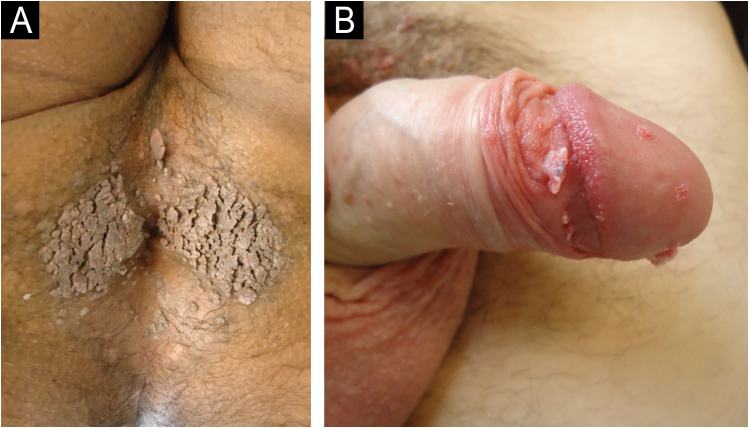
(A) Anal condyloma. (B) Penile condyloma.

**Fig. 2:**
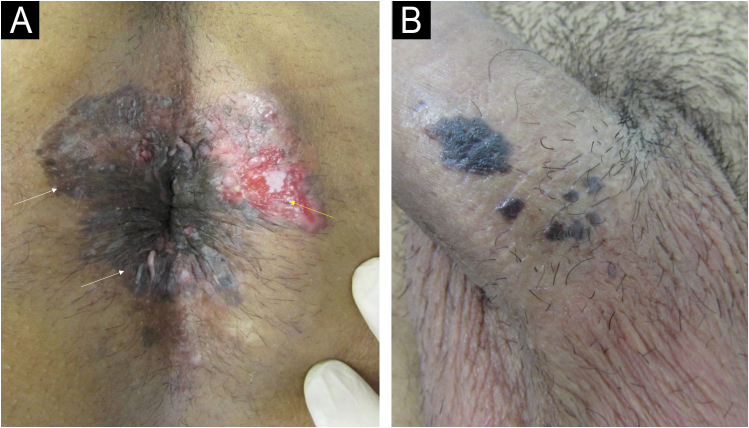
(A) Patient living with HIV using antiretroviral therapy with undetectable VL and CD4 327 cells/mm^3^ showing concomitant lesions caused by HPV: squamous cell carcinoma (SCC) *in situ*/Bowen's disease (white arrows) and invasive SCC (yellow arrow) in the perianal region. (B) Patient with follicular lymphoma undergoing chemotherapy with SCC *in situ*/Bowen's disease in the penis shaft.

**Fig. 3:**
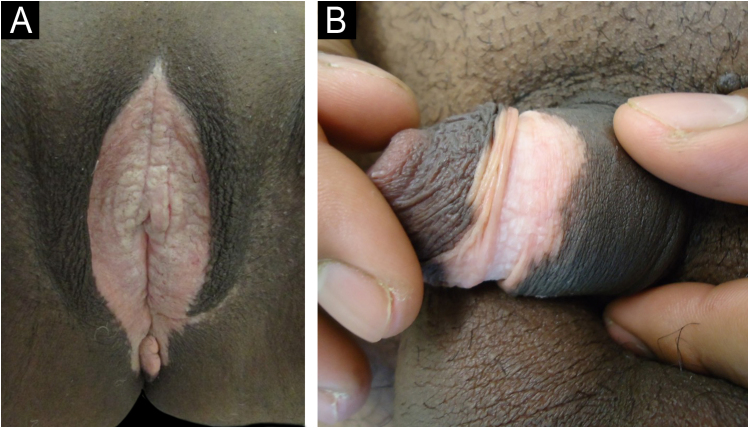
(A) Lichen sclerosus et atrophicus of the vulva (differential diagnosis of vitiligo). (B) Lichen sclerosus et atrophicus of the penis showing a ring of circumferential constriction in the center of the achromic lesion characteristic of the disease.

The significant number of consultations recorded in this study represents a relatively reliable scenario of the distribution of diagnoses of patients with anogenital complaints seeking public care in the city of São Paulo.

When analyzing the year-by-year distribution of consultations during the study period, a progressive reduction can be observed from 2013 onwards, with a marked decrease from 2020 to 2024, probably as a consequence of the COVID-19 pandemic.

The fact that the vast majority of consultations were related to the diagnosis of CA is consistent with data in the literature, as is the sequence of the other most prevalent diagnoses, syphilis, and herpes simplex. It is believed this epidemiological trend observed in a tertiary hospital may be related to the chronicity of CA, syphilis, and herpes simplex. More acute conditions, such as chancroid and anogenital discharge, were rarely reasons for consultation, probably because they were resolved by seeking medical care from primary care physicians or emergency rooms. The negative impact on the quality of life of patients with condyloma may also be another reason that makes them keep trying to seek medical care.^2^, ^6^, ^7^

It is important to note the significant number of diagnoses not related to STI, such as inflammatory and neoplastic diseases. Studies conducted in other countries show that inflammatory lesions are the most frequently diagnosed in clinics treating genital lesions.^3^, ^4^

It is also important to highlight that the number of patients treated for squamous cell carcinoma in the anogenital region took fourth place. This diagnosis is intrinsically related to the neoplastic potential of the HPV virus, which is also responsible for CA and is more aggressive in immunocompromised patients. In a study conducted in 2019 covering the entire national territory, it was observed that among the 6,388 healthy young adults assessed, the prevalence of general HPV was 53.6%. Of these, the majority (35.2%) had at least one high-risk HPV.^8^ These data show that not only is a large part of the population carrying this potentially oncogenic virus, but it is also following its pathogenic course, possibly in a significant fraction of this population. This fact reinforces the extreme importance of public measures focused on vaccination against HPV.

Although the data collection period included Mpox outbreaks that occurred worldwide – and therefore in Brazil^1^, ^2^ – there were no records of patients with Mpox lesions being treated due to structural impediments to the flow of care at the studied hospital.^9^, ^10^

The numbers presented herein represent, within their limitations, the reality of the different aspects involving anogenital dermatoses in the city of São Paulo and may serve as indicators for the development of public health policies, especially those regarding STI.

## Authors' contributions

Julia Aires Thomaz Maya: Collection of data, or analysis and interpretation of data; drafting and editing of the manuscript or critical review of important intellectual content; collection, analysis and interpretation of data; critical review of the literature; approval of the final version of the manuscript.

Kaique Arriel: Collection of data, or analysis and interpretation of data; drafting and editing of the manuscript or critical review of important intellectual content; collection, analysis and interpretation of data; critical review of the literature; approval of the final version of the manuscript.

Laura Stamato de Figueiredo: Collection of data, or analysis and interpretation of data; drafting and editing of the manuscript or critical review of important intellectual content; collection, analysis and interpretation of data; critical review of the literature; approval of the final version of the manuscript.

John Verrinder Veasey: Design and planning of the study; collection of data, or analysis and interpretation of data; statistical analysis; drafting and editing of the manuscript or critical review of important intellectual content; collection, analysis and interpretation of data; effective participation in research orientation; intellectual participation in the propaedeutic and/or therapeutic conduct of studied cases; critical review of the literature; approval of the final version of the manuscript.

## Financial support

None declared.

## Conflicts of interest

None declared.
